# Surgical treatment of neurocysticercosis. Retrospective cohort study and an illustrative case report: Tratamento cirúrgico da neurocisticercose. Estudo de coorte retrospectivo e um caso ilustrativo

**DOI:** 10.1590/1516-3180.2016.0304171216

**Published:** 2017-04-03

**Authors:** Aline Lariessy Campos Paiva, João Luiz Vitorino Araujo, Vinicius Ricieri Ferraz, Renan Maximilian Lovato, Charles Alfred Grander Pedrozo, Guilherme Brasileiro de Aguiar, José Carlos Esteves Veiga

**Affiliations:** I MD. Resident, Discipline of Neurosurgery, Faculdade de Ciências Médicas da Santa Casa de São Paulo (FCMSCSP), São Paulo (SP), Brazil.; II PhD. Attending Neurosurgeon, Discipline of Neurosurgery, Faculdade de Ciências Médicas da Santa Casa de São Paulo (FCMSCSP), and Neurosurgeon at Arnaldo Vieira de Carvalho Cancer Institute, Oncocenter and Hospital Nove de Julho, São Paulo (SP), Brazil.; III MSc. Attending Neurosurgeon, Discipline of Neurosurgery, Faculdade de Ciências Médicas da Santa Casa de São Paulo (FCMSCSP), São Paulo (SP), Brazil.; IV PhD. Full Professor and Head, Discipline of Neurosurgery, Faculdade de Ciências Médicas da Santa Casa de São Paulo (FCMSCSP), São Paulo (SP), Brazil.

**Keywords:** Neurocysticercosis, Hydrocephalus, Epilepsy, Ventriculoperitoneal shunt, Central nervous system infections, Neurosurgical procedures, Case reports, Cohort studies

## Abstract

**CONTEXT AND OBJECTIVE:**

Neurocysticercosis is prevalent in developing countries and manifests with several neurological signs and symptoms that may be fatal. The cysts may be parenchymal or extraparenchymal and therefore several signs and symptoms may occur. Depending on their location, neurosurgical procedures may be required, sometimes as emergencies. The aim here was to review 10-year statistics on all surgical neurocysticercosis cases at a large public tertiary-level hospital.

**DESIGN AND SETTING:**

Retrospective cohort at a large public tertiary-level hospital.

**METHODS:**

All surgical neurocysticercosis cases seen between July 2006 and July 2016 were reviewed. Parenchymal and extraparenchymal forms were considered, along with every type of surgical procedure (shunt, endoscopic third ventriculostomy and craniotomy). The literature was reviewed through PubMed, using the terms “neurocysticercosis”, “surgery”, “shunt” and “hydrocephalus”.

**RESULTS:**

37 patients underwent neurosurgical procedures during the study period. Most were male (62.16%) and extraparenchymal cases predominated (81%). Patients aged 41 to 50 years were most affected (35.13%) and those 20 years or under were unaffected. Ventricular forms were most frequently associated with hydrocephalus and required permanent shunts in most cases (56.57%).

**CONCLUSIONS:**

The treatment of neurocysticercosis depends on the impairment: the parenchymal type usually does not require surgery, which is more common in the extraparenchymal form. Hydrocephalus is a frequent complication because the cysts often obstruct the cerebrospinal flow. The cysts should be removed whenever possible, to avoid the need for permanent shunts.

## RESUMO

**CONTEXTO E OBJETIVO:**

A neurocisticercose é prevalente em países em desenvolvimento e manifesta-se com vários sinais e sintomas neurológicos que podem ser fatais. Os cistos podem ser parenquimatosos ou extraparenquimatosos, portanto vários sinais e sintomas podem estar presentes. Dependendo da sua localização, procedimentos neurocirúrgicos podem ser necessários, às vezes em caráter emergencial. O objetivo foi revisar dados estatísticos de um período de 10 anos de todos os casos cirúrgicos de neurocisticercose num grande hospital público terciário.

**TIPO DE ESTUDO E LOCAL:**

Coorte retrospectiva de um grande hospital público terciário.

**MÉTODOS:**

Todos os casos cirúrgicos de neurocisticercose de pacientes tratados entre julho 2006 e julho 2016 foram revisados. As formas parenquimatosas e extraparenquimatosas foram consideradas, assim como tipo de procedimento cirúrgico (derivação, terceiroventriculostomia endoscópica e craniotomia). A literatura foi revisada por meio da PubMed, utilizando-se os termos “neurocysticercosis”, “surgery”, “shunt” e “hydrocephalus”.

**RESULTADOS:**

37 pacientes foram submetidos a procedimentos neurocirúrgicos nesse período, a maioria do sexo masculino (62.16%%) e casos extraparenquimatosos predominaram (81%). Pacientes com idade 41-50 anos foram os mais afetados (35,13%) e aqueles com 20 anos ou menos não foram afetados. As formas ventriculares mais frequentemente estiveram associadas a hidrocefalia e necessitaram da realização de *shunts* definitivos na maior parte dos casos (56,57%).

**CONCLUSÕES:**

O tratamento depende da forma de acometimento: o tipo parenquimatoso usualmente não necessita de cirurgia que é mais comum na forma extraparenquimatosa. Hidrocefalia é uma complicação frequente pois muitas vezes os cistos obstruem o fluxo liquórico. A remoção dos cistos deve ser realizada sempre que possível para evitar a necessidade de derivações definitivas.

## INTRODUCTION

Neurocysticercosis is caused by central nervous system (CNS) infection due to *Taenia solium* (pork tapeworm) larvae.^1^ It constitutes the most common cause of epilepsy^1^ and hydrocephalus in adults who live in developing countries.^1^^,^^2^ This disease may be acquired when a healthy person ingests eggs from the feces of a tapeworm carrier through contaminated water or vegetables. The infection may affect the brain parenchyma (in some cases, it may mimic brain tumors^3^) or it may be extraparenchymal, in the cisternae, subarachnoid space or intraventricular areas. The most frequent location is in the cerebral hemispheres,^1^^,^^4^ where lesions are initially surrounded by edema and subsequently calcify but remain as epileptic foci.

The incidence of this disease is greater in developing countries, although some large studies have shown increasing incidence in developed countries such as the United States,^5^ and it is very variable around of the world. In Latin America, the incidence also varies depending on the urban or rural region, from 121.7 to 138.4 cases per 100,000 individuals per year.^6^ Sanitary conditions have a close relationship with neurocysticercosis, and combating this disease is a priority for the World Health Organization (WHO).^2^ It is one of the seven neglected endemic zoonoses targeted by WHO. The clinical manifestations of neurocysticercosis have been well known since the late 1800s and early 1900s.^7^ Cysticerci in the CNS can cause several neurological manifestations, depending on the cyst location and stage and the numbers of cysts.^4^

The treatments include use of antiparasitic drugs, especially praziquantel and albendazole.^4^ However, when ventricular or cisternal forms are present, these drugs are not effective. Either albendazole or praziquantel is effective for killing live cysticerci. Albendazole is currently the drug of choice because of its slightly greater efficacy, better availability and lower cost. It is also very important to treat symptoms such epilepsy using antiepileptic drugs (AEDs). A neurosurgical approach is usually required for ventricular forms, which cause hydrocephalus, and also in cases in which the cerebrospinal fluid (CSF) flow is altered in other regions such as in the cisternae spaces.^4^^,^^8^

The prognosis for neurocysticercosis is usually good when timely treatment is instituted. In neurosurgical approaches, the initial aim should be to try to withdraw obstructions, and thus to remove cysts when possible. Sequelae such as adult epilepsy are common and these patients usually require long-term use of AEDs, with follow-up from the infectious disease team.^4^^,^^8^

## OBJECTIVE

The aim of this study was to describe all the neurosurgical cases (parenchymal and extraparenchymal forms) seen at our institution over the past 10 years, focusing on cisternal impairment.

## METHODS

This was an observational longitudinal and retrospective study in which all patients with a diagnosis of neurocysticercosis who underwent any surgical procedure were included. A database was constructed to analyze all neurosurgical cases of neurocysticercosis that were seen at a large tertiary-level hospital in São Paulo, Brazil, over the past ten years (July 2006 to July 2016). The analysis took gender, age, type of impairment (parenchymal or extraparenchymal), presence of hydrocephalus, type of neurosurgical approach proposed and patient’s origin (rural or urban area) into consideration.

In addition, a cerebellomedullary case is reported in greater detail, with the clinical picture and intraoperative images.

A detailed review of the literature was also conducted, focusing on neurosurgical approaches towards this disease. An extensive search was performed in PubMed using the terms: “neurocysticercosis”, “surgery”, “shunt” and “hydrocephalus”.

## RESULTS

A retrospective analysis was conducted on 37 patients who underwent neurosurgical procedures to treat neurocysticercosis over a ten-year period (2006-2016).

The male gender was more affected (62.16%). Patients between 41 and 50 years were most affected (35.13%), followed by the age groups from 31 to 40 years (21.62%), from 21 to 30 years (16.21%) and finally from 51 to 60 and over 61 years (13.51% each). Children (20 years and under) were unaffected in this sample (Table 1). Patients coming from rural areas were clearly more affected (75.67%).

**Table 1:**
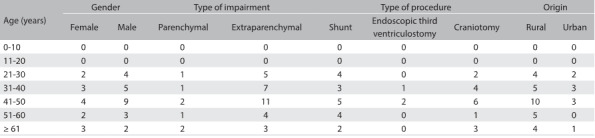
Variables considered for epidemiological analysis on 37 consecutive surgical neurocysticercosis cases

The extraparenchymal type predominated, accounting for 81% of all cases. Among these patients, 76% had only the ventricular form, 14% only the cisternal form and 10% both forms. 

Regarding neurosurgical approaches, 8% required endoscopic intervention and the other cases were equally divided between craniotomy (46%) and ventriculoperitoneal shunt (VPS) (46%). The procedure depended on the type of impairment, as shown in Table 2. For example, the parenchymal form required craniotomy in most cases (71.43%).

**Table 2:**
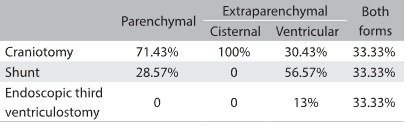
Type of procedure versus form of neurocysticercosis, considering all 37 patients.

**Figure 1:**
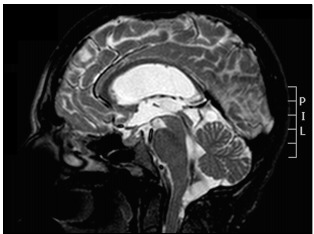
Preoperative sagittal T2-weighted magnetic resonance imaging (MRI) showing several cysts in cerebellomedullary cisterna obstructing cerebrospinal fluid flow, and showing hydrocephalus.

**Figure 2:**
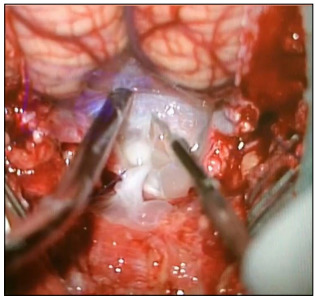
Microsurgical appearance of cerebellomedullary neurocysticercosis.

An illustrative case of a 31-year-old female patient who presented with epilepsy and signs and symptoms of intracranial hypertension (progressive headache and papilledema) is described here. Complementary investigation revealed eosinophilic meningitis and neuroimaging investigation showed hydrocephalus and cystic lesions in the cerebellomedullary cisterna (Figure 1). Suboccipital craniotomy to excise the cysts was proposed (Figure 2). After the cisterna had been opened, several cysts that were obstructing the CSF flow could be seen (Figure 2).

## DISCUSSION

Neurocysticercosis is a CNS infection in which the incidence is closely related to sanitary conditions. For this reason, it is more prevalent in developing countries^1^^,^^2^ and especially in rural areas of these countries. It is caused by the larval form of *Taenia solium*. Humans are usually the definitive host, but in some cases, the cycle becomes altered and fecal-oral contamination occurs. In these cases, humans are the intermediate host.^1^^,^^2^^,^^4^

The cysts may be found in almost any organ or tissue. In most locations, they will not be noticed, but when cysts are located in the CNS, many symptoms may be present and may produce severe and disabling disease, which is sometimes lethal.^8^ Because the cysts are initially surrounded by significant degrees of edema, this constitutes an important epileptogenic factor.^4^ Even after degeneration of the cysticercus, the irritant remains and the patient might become epileptic.^9^ Therefore, epilepsy is the most frequent symptom of the parenchymal type.^1^^,^^9^ In the extraparenchymal form, in which an obstruction occurs at some point of the CSF flow (Figures 1 and 2), patients may present signs and symptoms of intracranial hypertension secondary to hydrocephalus. In one of the cases analyzed in the present study, a patient had an initial presentation of aphasia: she had perisylvian cysts and the initial edema could have affected the speech area. Brain neoplasm was initially considered as primary differential diagnosis^3^ and this case was previously reported.^3^

It was observed that patients could present with several different clinical pictures caused by the presence of brain cysts, depending on their locations. The severity of the cases was very variable, from asymptomatic to extremely symptomatic with elevated intracranial pressure and a comatose state.

Two main types of neurocysticercosis have been defined, depending on its location: parenchymal or extraparenchymal.^1^^,^^3^^,^^10^ To institute the correct therapeutic approach, it is essential to differentiate these types. Parenchymal cysticercosis generally has a better prognosis, mainly because it tends to respond to antiparasitic drugs: albendazole (15 mg/kg/day) or praziquantel (50-75 mg/kg/day) for 15 days. During their use, concomitant use of steroids has been advocated,^10^ without any need for neurosurgical treatment.^4^^,^^11^ Surgical intervention is required when this lesion in its racemose form, which leads to a significant mass effect with edema surrounding it. In a few cases, this lesion can mimic a brain tumor (as suggested from neuroimaging),^3^ as in one of the cases evaluated in this review. Such lesions are more often associated with epileptic manifestations.^10^

On the other hand, the extraparenchymal form may present as cysts in the subarachnoid and cisternal spaces and intraventricular areas. This is more often related to severe symptoms such as intracranial hypertension (secondary to hydrocephalus due to obstruction of CSF circulation). It is also associated with marked inflammation and increased concentrations of proteins and cells in the CSF, resulting from continued exposure to remnants of parasite membranes.^4^^,^^10^

The treatment for this type is essentially neurosurgical, with cyst removal by means of endoscopy or craniotomy, depending on their locations.^9^ For example, in the case reported in Figure 1, it was decided to use suboccipital craniotomy, to provide complete resection of the cerebellomedullary cysts. Patients may not require permanent shunts such as ventriculoperitoneal valves. The basic principle is to remove the obstruction through removing cysts and adhesions to facilitate CSF flow. However, despite cyst removal, some patients might still require permanent shunts. This could be due to chronic inflammatory processes caused by the parasite.^6^ Initially, the patient whose case is reported in Figure 1 did not require a shunt, but about two weeks after the first surgery, she presented with hydrocephalus and a definitive shunt procedure was performed.

In the present case series, diverging from most studies in the literature,^1^^,^^4^^,^^6^^,^^8^ the extraparenchymal form predominated. This may have been because the present study only reported on neurocysticercosis cases that required operations. The parenchymal form, which is generally the most common type, usually does not require neurosurgical intervention, unlike cases in which the disease has an extraparenchymal location.

## CONCLUSION

It may present with several signs and symptoms, and therefore the diagnosis is made through clinical examination and neuroimaging (epidemiological factors may also help). A neurosurgical approach is usually required in cases of the extraparenchymal form. Thus, when only surgical cases are considered, the incidence of this type is greater, as in the present review. The aim of surgical procedures should always be to remove the cysts and avoid the need for permanent shunts when hydrocephalus is present. These patients should be followed up for indefinite periods of time, given that many of them remain epileptic after the acute phase.
